# Treating childhood pneumonia in hard-to-reach areas: A model-based comparison of mobile clinics and community-based care

**DOI:** 10.1186/1472-6963-12-9

**Published:** 2012-01-10

**Authors:** Catherine Pitt, Bayard Roberts, Francesco Checchi

**Affiliations:** 1 Department of Global Health & Development, London School of Hygiene & Tropical Medicine, 15-17 Tavistock Place, London WC1H 9SH, UK; 2 Department of Health Services & Policy Research, London School of Hygiene & Tropical Medicine, 15-17 Tavistock Place, London WC1H 9SH, UK; 3 Department of Disease Control, London School of Hygiene & Tropical Medicine, Keppel Street, London WC1E 7HT, UK

**Keywords:** Pneumonia, treatment, mobile clinic, community health workers, decision model, crisis, humanitarian, remote, rural

## Abstract

**Background:**

Where hard-to-access populations (such as those living in insecure areas) lack access to basic health services, relief agencies, donors, and ministries of health face a dilemma in selecting the most effective intervention strategy. This paper uses a decision mathematical model to estimate the relative effectiveness of two alternative strategies, mobile clinics and fixed community-based health services, for antibiotic treatment of childhood pneumonia, the world's leading cause of child mortality.

**Methods:**

A "Markov cycle tree" cohort model was developed in Excel with Visual Basic to compare the number of deaths from pneumonia in children aged 1 to 59 months expected under three scenarios: 1) No curative services available, 2) Curative services provided by a highly-skilled but intermittent mobile clinic, and 3) Curative services provided by a low-skilled community health post. Parameter values were informed by literature and expert interviews. Probabilistic sensitivity analyses were conducted for several plausible scenarios.

**Results:**

We estimated median pneumonia-specific under-5 mortality rates of 0.51 (95% credible interval: 0.49 to 0.541) deaths per 10,000 child-days without treatment, 0.45 (95% CI: 0.43 to 0.48) with weekly mobile clinics, and 0.31 (95% CI: 0.29 to 0.32) with CHWs in fixed health posts. Sensitivity analyses found the fixed strategy superior, except when mobile clinics visited communities daily, where rates of care-seeking were substantially higher at mobile clinics than fixed posts, or where several variables simultaneously differed substantially from our baseline assumptions.

**Conclusions:**

Current evidence does not support the hypothesis that mobile clinics are more effective than CHWs. A CHW strategy therefore warrants consideration in high-mortality, hard-to-access areas. Uncertainty remains, and parameter values may vary across contexts, but the model allows preliminary findings to be updated as new or context-specific evidence becomes available. Decision analytic modelling can guide needed field-based research efforts in hard-to-access areas and offer evidence-based insights for decision-makers.

## Background

As of 2010, fewer than one-third of the 68 priority countries with the highest levels of child mortality were on track to meet the Fourth Millennium Development Goal (MDG) of reducing mortality in children aged under five by two-thirds between 1990 and 2015. In many countries where overall progress has been made, that progress has been inequitable, with higher mortality among children in marginalised regions, especially remote areas and those affected by armed conflict [1,2]. In such settings, the leading causes of childhood deaths are pneumonia, diarrhoea, neonatal causes, and (where present) malaria [3-5].

Humanitarian relief agencies often provide services through mobile clinics to serve populations living beyond the reach of existing facilities. These mobile clinics often operate in chronic crises, where security is poor, logistics are difficult, and mortality is high. An increasing number of people affected by complex emergencies now live spread out across large and remote geographical areas, in places such as Somalia, Afghanistan, the Central African Republic, and Southern Sudan [3]. They are consequently harder to reach with humanitarian interventions and suffer prolonged excess mortality [3]. There is strong evidence that these mobile clinics, which usually provide relatively skilled services on an intermittent basis, can make a positive health impact with preventive activities such as vaccinations, hygiene promotion, and antenatal counselling, as well as through certain curative services for chronic or slow-onset diseases such as intestinal parasites, guinea worm, leishmaniasis, onchocerciasis, and trypanosomiasis [6-9]. In many cases, however, mobile clinics also focus on curative care for rapid-onset illnesses, such as malaria, pneumonia, and diarrhoea [10].

There is also growing consensus around the effectiveness and safety of community-based management of pneumonia by supporting low-skilled health workers to provide oral antibiotics in low-income and remote areas [11-14]. A meta-analysis of nine trials in seven countries by Sazawal *et al *found that community antibiotic treatment strategies resulted in an average 24% reduction in total under-five mortality and a 36% reduction in pneumonia mortality in under-fives in high mortality settings [11]. In Nepal, one of the priority countries on track to meet MDG4 [2], female community health volunteers (FCHV) save an estimated 6000 child lives each year by providing oral antibiotics for non-severe pneumonia in remote mountain communities. Despite being only semi-literate, Nepal's FCHVs have shown "minimal" evidence of antibiotics misuse, and 30,007 case reviews in 2005-6 revealed "no evidence of emerging co-trimoxizole resistance" [12].

Despite this evidence, community-based management of pneumonia is not standard practice amongst relief agencies in less stable conflict-affected situations, and the most commonly used guidelines for humanitarian settings are designed primarily for physicians and nurses [15,16]. Relief agencies train low-skilled community health workers (CHWs) largely as health promoters and instruct them to recommend referral for children needing antibiotic treatment [15]. However, CHW referral recommendations often do not lead to action even in stable settings, and CHWs unable to provide medication often lack credibility [12]. Indeed, even trained health workers often fail to convince families to take a child with severe complications to hospital in a timely manner [17,18]. Insecurity reinforces those factors that prevent many families from seeking care in stable environments; financial resources shrink, security concerns increase, travel is restricted, and heightened social tensions may change perceptions of the acceptability of seeking care from particular facilities or individuals. Relief agencies, donors, ministries of health, and other health actors should therefore consider how community case management of childhood pneumonia may compare with mobile clinics and other strategies they employ to reduce child mortality in hard-to-access populations.

This paper aims to generate quantitative evidence for health policy regarding the relative effectiveness of two alternative strategies, mobile clinics and CHWs in fixed posts, for antibiotic treatment of childhood pneumonia in hard-to-access populations. It develops a mathematical decision model that reflects both clinical aspects of pneumonia case management and practical aspects of implementing each intervention in order to provide recommendations to humanitarian agencies, donors, and other key decision-makers.

## Methods

A decision analytic model was developed according to the stages recommended by Briggs *et al *by specifying the decision problem and boundaries of analysis, structuring the decision model, identifying appropriate evidence, and dealing with uncertainty and heterogeneity [19].

### Decision problem

The study population was identified as a hypothetical cohort of children aged 1-59 months dispersed across a high-mortality, remote setting, where antibiotic treatment for pneumonia is currently unavailable. No geographic location was specified.

Outcomes for this hypothetical cohort were examined under the following three scenarios:

1) No pneumonia treatment available;

2) Mobile clinics are present in the community on an intermittent basis, providing highly accurate diagnosis and treatment when they are present and no treatment when they are absent;

3) A CHW is paid and supported to diagnose and treat pneumonia with antibiotics in the community; while living in the community and therefore available at virtually any time in locations as accessible (if not more so) than mobile clinics, the CHW provides less accurate diagnosis and treatment of pneumonia than the mobile clinic staff.

The model predicts the cumulative number of pneumonia deaths over a given period. Accordingly, the comparison between the two strategies is expressed as the relative rate of death from pneumonia under either strategy, compared to no treatment. While reducing the number of cases that become severe and the duration of disease may also improve nutritional status, development and educational attainment, and generally mitigate other untoward outcomes [20], these potential secondary benefits were not included in the model.

### Model structure

We adopted a static rather than transmission dynamic model, as the strategies evaluated are likely to have a negligible impact on disease transmission. The vast majority of children in developing countries are infected with *Streptococcus pneumoniae, Haemophilus influenzae *type B and other aetiologic agents of life-threatening pneumonia: such nasopharyngeal carriage is mostly asymptomatic and correlates poorly with invasive disease [21,22]. The incidence of pneumonia seems to depend mainly on host and environmental factors [22,23].

Using Microsoft Excel Visual Basic [24], we developed a modified Markov cycle tree model [19,25] to reflect the stochastic nature of and uncertainty in various processes determining childhood pneumonia outcome under each of the scenarios. Markov models are characterised by a finite number of discrete health states, with individuals facing a probability of transition from one health state to another at the end of each of a series of discrete time steps (in our case days). The model was implemented over a time horizon of one hundred days, which reflects the short-term nature of humanitarian programme implementation.

#### Health states

**Figure 1:**
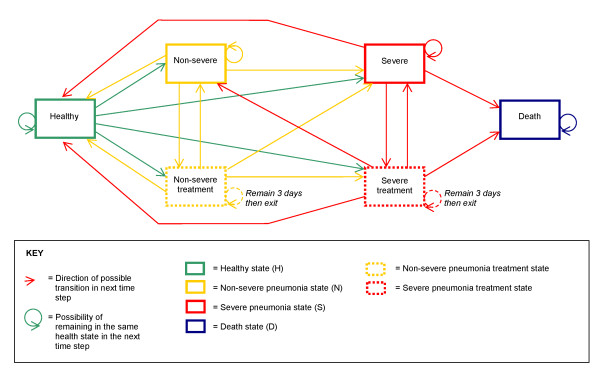
**Simplified model structure**.

The six health states are healthy (meaning not ill with pneumonia), non-severe pneumonia, non-severe pneumonia under treatment, severe pneumonia, severe pneumonia under treatment, and death (Figure 1). Children enter the model in the healthy state, and are exposed to a constant pneumonia incidence rate, which reflects our assumption that case management will not affect incidence.

#### Tunnel states and memory

The so-called "Markov assumption" or "memoryless" aspect of the traditional Markov model was relaxed by creating "tunnel states." This approach built temporary memory into the model by tracking the first five days spent with pneumonia. For example, a healthy child who becomes ill with non-severe pneumonia for two days and then develops severe pneumonia on the third day moves through the health states "non-severe day 1," "non-severe day 2," and "severe day 3" (Additional file 1). Incorporating memory into the model allowed for the calculation of time-dependent rates of care-seeking described below.

#### Transition probabilities

**Figure 2:**
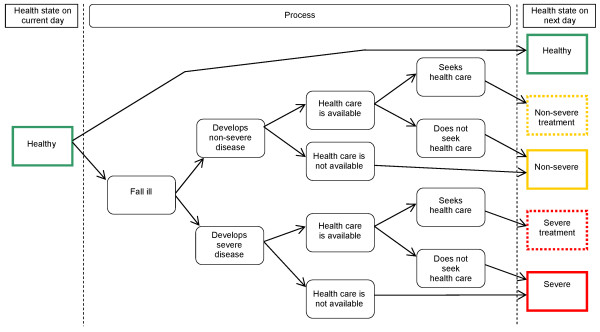
**Decision tree: transitions from the healthy state**. Healthy children may remain healthy or transition to any of the disease states on the following day. If a child develops pneumonia and health care is both available and sought, the child transitions directly to treatment for pneumonia; otherwise, the child transitions to a disease state (non-severe or severe pneumonia) without treatment. Values and equations defining transition probabilities are detailed in the Appendix.

Figure 2 depicts the possible daily transitions for a healthy child. If a child develops pneumonia, health care is available and the child's caregiver seeks treatment within the same day, the child may transition directly from the healthy to the non-severe or severe treatment states. The decision tree is virtually identical for children starting the day in a non-healthy state, except that the transitions for a child with severe pneumonia are restricted to remaining severe, recovery or death. Decision trees and equations used to calculate transition probabilities are provided in Additional file 1.

#### Care-seeking behaviour

Studies indicate that the rate of care seeking is time-dependent, with no reported differences between non-severe and severe pneumonia [15,26-28]. Accordingly, the daily probability of seeking care was modelled as a function of how many days the child had spent with pneumonia, irrespective of severity. Available evidence was used to calculate the relative rate of care seeking for each day a child may have pneumonia, up to a maximum of five days, beyond which data are not available and the relative rates are therefore modelled as remaining constant.

### Parameter values and sensitivity analyses

Parameter values were informed by evidence from the literature and expert advice. Ethical approval was obtained for expert interviews from the London School of Hygiene and Tropical Medicine. PubMed, EMBASE and Google Scholar were searched for key terms and authors; literature recommendations were sought during expert interviews; and the bibliographic trail of relevant references was followed to exhaustion. Sources of parameter values are provided in Additional file 1, and the values of key parameters are shown in Table 1.

**Table 1:** Values of key model parameters in the baseline analysis

Parameter		Scenario		Distribution	Source
			
	No treatment	Mobile clinic	Community Health Worker		
Incidence of pneumonia (episodes per child per year)	0.7	0.7	0.7	Beta	Rudan et al. [30]

Care-seeking behaviour	n/a	Median duration before care sought = 3 days, Cumulative probability of seeking care = 90%		Lognormal	Kallander et al [26] Sodemann et al [45]

Probability that treatment is available on any given day	0%	100% on day of weekly visit, 0% on other days	100%	n/a	Assumption of the model

Probability of correct diagnosis and prescription	n/a	90%	80%	Beta	Kallander et al [27] Dawson et al [12] Lim et al [42]

Probability of adherence to treatment	n/a	80%		Beta	Checchi et al [41]

Probability that treatment for non-severe pneumonia is efficacious	n/a	95%		Beta	Hazir et al [13] Lim et al [42]

Probability that treatment for severe pneumonia is efficacious	n/a	90%	80%	Beta	Kabra et al [14] Lim et al [42] Zaman et al [46] Johnson et al [47] Hazir et al [13] Banajeh et al [48]

To account for uncertainty in parameter values, we implemented the model stochastically. Accordingly, beta and lognormal probability distributions for each parameter were constructed following the standard methods proposed by Briggs *et al *[19], ensuring that the distributions reflected reasonable beliefs about the parameter and were defined over an appropriate interval. The standard error for a parameter value was set to five percent of the mean value of that parameter [29]. We then ran 10,000 iterations of the model, sampling randomly from each parameter distribution at each time step.

We also performed sensitivity analyses to explore how results would vary according to pneumonia incidence rate (the sensitivity range of 0.3 to 1.9 pneumonia episodes per child-year was based on Rudan *et al *[30]: the lower limit corresponds with Rudan's estimate of the median in developing countries, while the upper limit corresponds to the maximum value recorded in 28 community-based longitudinal studies that met quality criteria); the accuracy of triage; the frequency of mobile clinic visits; and care-seeking behaviour.

## Results

### Validity

In the no treatment scenario, the model predicted a pneumonia-specific mortality rate of 0.51 (95% credible interval or CI: 0.49 to 0.54) pneumonia deaths per 10,000 child-days, or 18.62 (95% CI: 17.89 to 19.71) deaths per 1000 child-years. Assuming pneumonia causes 23% of child mortality [31], this translates to an all-cause mortality rate of 2.24 deaths per 10,000 child-days or 71.25 deaths per 1000 child-years. Such rates are consistent with the high mortality settings addressed in this paper, as the commonly agreed thresholds defining a humanitarian emergency are 2.1 deaths in Sub-Saharan Africa or 1.7 deaths in least developed countries per 10,000 child-days [32].

### Baseline analysis

For the mobile clinic strategy, baseline analysis predicted a median pneumonia-specific mortality rate of 0.45 deaths per 10,000 child-days (95% CI: 0.43 to 0.48), or a reduction of 12.1%. The CHW strategy produced a lower median rate of 0.31 deaths per 10,000 child-days (95% CI: 0.29 to 0.32), or a 40.5% reduction. This latter figure is comparable to the pneumonia-specific mortality reductions measured in CHW projects in Uganda by Kallander *et al *[27] and in Nepal by Dawson *et al *[12]. While all iterations of the mobile clinic strategy resulted in mortality reductions with respect to no treatment, all iterations of the CHW strategy resulted in greater mortality reductions than the mobile clinic strategy. The CHW strategy is therefore superior to a weekly mobile clinic under baseline assumptions.

### Sensitivity analyses

**Figure 3:**
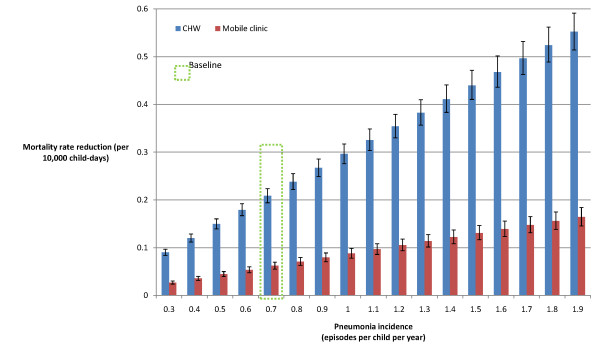
**Sensitivity analysis: variation in pneumonia incidence**.

**Figure 4:**
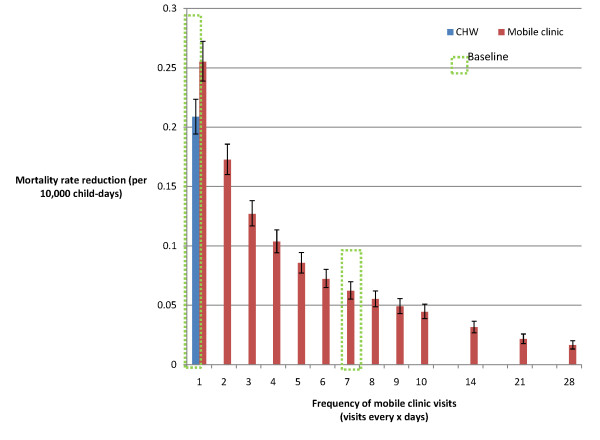
**Sensitivity analysis: variation in mobile clinic frequency**.

**Figure 5:**
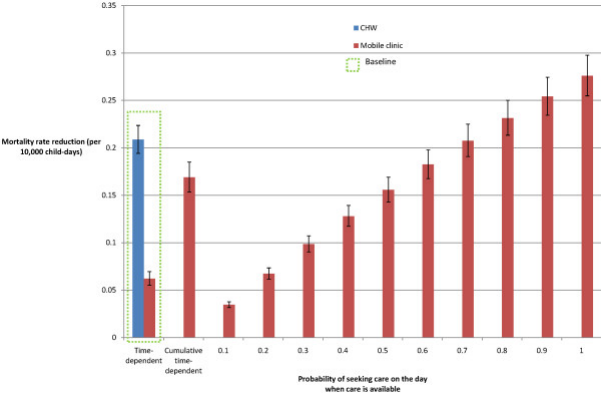
**Sensitivity analysis: variation in health care seeking behaviour**.

As pneumonia incidence increased, the CHW strategy achieved increasingly greater mortality reductions than the mobile clinic strategy (Figure 3). However, mobile clinics that visit every day achieved greater mortality reductions than the baseline CHW strategy (Figure 4), as would be expected given the former's higher diagnostic accuracy and lower case-fatality ratio (CFR) for severe cases. If the mobile clinic were to diagnose and correctly prescribe treatment for pneumonia with 100% sensitivity, it would achieve a 12% greater pneumonia-specific mortality reduction than under the baseline assumption of 90% sensitivity (Figure 5). If the CHW strategy were only to diagnose and prescribe treatment correctly for 40% of cases seen rather than the baseline assumption of 80%, it would achieve a 52% lower mortality reduction of 0.1 deaths per 10,000 child-days, which would be approximately equivalent to a mobile clinic with 90% sensitivity visiting every four days (Figure 4).

**Figure 6:**
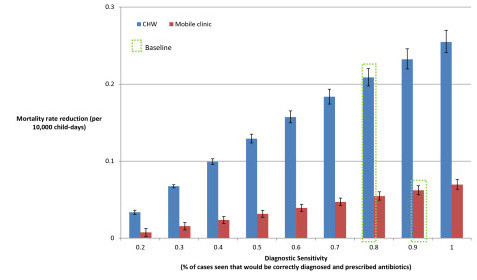
**Sensitivity analysis: variation in sensitivity of diagnosis and prescription**.

Assumptions regarding the time-dependent nature of care-seeking were also explored. Caregivers who know on which days the mobile clinic will visit may adapt their care-seeking behaviour; instead of depending on time since illness onset, care-seeking may simply be a binary probability on the day of clinic visit. Figure 6 shows that the mortality reduction achieved by a weekly mobile clinic would be comparable to those of a CHW if approximately 70% of children with any severity of pneumonia onset were taken to the mobile clinic and the mobile clinic were able to consult all cases. In addition, if all caregivers who would have sought care on a day prior to the mobile clinic's visit did so on the day of the visit (and whose careseeking behaviour was therefore a cumulative time-dependent probability), the mobile clinic would reduce mortality by 0.17 deaths per 10,000 child-days (CI: 0.15 to 0.19) and achieve a 32.8% reduction in pneumonia-specific mortality.

## Discussion

We find that a CHW in a fixed health post would achieve greater mortality reductions than a weekly mobile clinic strategy in the majority of sensitivity analyses conducted. In univariate sensitivity analyses, mobile clinics achieved greater mortality reductions where the probability of seeking care from a mobile clinic was significantly higher than from a CHW, and where mobile clinics visited communities daily. Mobile clinics would also achieve greater mortality reductions than the CHW strategy if the mobile clinic had 100% sensitivity to diagnose and treat children while the CHW strategy had approximately 30% or lower sensitivity, or if a number of variables simultaneously differed substantially from our baseline assumptions. For a number of important parameters, only limited evidence is available and parameter values may differ between contexts. These findings therefore do not conclusively show one strategy to be more effective than the other. Rather, current evidence indicates that a CHW in a fixed post is a viable alternative to mobile clinics, and therefore warrants consideration in high-mortality, hard-to-access areas.

In line with humanitarian decision-making, this model focussed on the short term impact of the alternative strategies; however, the longer term impact on health and health systems should also be taken into account. Mobile clinics may be quicker to implement in response to a sudden-onset crisis, but they may also risk creating dependence and undermining community coping strategies [33]. Supporting CHWs may foster sustainable local development and be more readily integrated into the public health system, but identifying potential CHWs is not always straightforward [34], especially when social tensions are high and different individuals are acceptable to different groups. Both strategies rely upon effective and reliable drugs supply and CHWs especially require regular supervision and support to be effective [12,34].

Agencies with the resources to respond quickly to an emerging crisis by deploying mobile clinics should consider initiating a transition to community-based treatment from their very first visit by identifying existing or potential CHWs to train. The role of the mobile clinic can then evolve into providing regular high-quality supplies and supervision to CHWs. With preparation, this supportive approach can also ensure that treatment remains available even if insecurity or other obstacles prevent the mobile team from visiting for a period. By supporting CHWs to treat pneumonia and other acute diseases, the mobile team could potentially also use its visits to engage in preventive interventions, such as immunizations, and to provide specialized treatment for more chronic conditions.

By providing a direct quantitative comparison of the health impact of these two strategies for reaching hard-to-access populations, this paper responds to calls from leaders in epidemiology and public health for research into implementation strategies in high-mortality settings [1,35,36]. Modelling can be a useful tool to foster rational decision-making based on explicit assumptions and scientific evidence for hard-to-access populations in low-income areas. While computationally intensive, the model is transparent and intuitive, and the decision analytic approach is adapted to the needs of decision-makers. As new data comes to light, parameter values can be changed and aspects of the model can be refined.

In quantifying the uncertainty in the decisions, the model also highlights the need for humanitarian and development programmes to undertake research in hard-to-access areas to reduce decision uncertainty. Improving the evidence base would require focussed data collection on parameter values from a given setting where both strategies are implemented or formal comparisons of the two approaches, although the latter could potentially have untenable ethical implications. Cost-effectiveness analyses would offer a more complete evidence base for deciding between the two strategies, as even in emergency interventions, efficiency is required to maximize the impact of the finite human, logistic, and financial resources that can be mobilized. Although the relative costs of the interventions are likely to vary considerably according to the specificities of each context, in general mobile clinics have been a resource-intensive intervention [10]; this suggests that a CHW strategy will usually be the more cost-effective option of the two if local contextual parameters indicate that the two strategies would have similar curative effectiveness.

This model can and should also be extended to the treatment of other key childhood diseases. In addition to diarrhoea and malaria, neonatal deaths constitute a significant proportion of under five mortality and many are attributable to invasive bacterial infections, including pneumonia, sepsis, and meningitis, which can be treated with antibiotics [2,35,37,38]. Sazawal *et al *provide some evidence for community management of neonatal pneumonia [11], and a number of countries have begun to include protocols for neonatal care within IMCI guidelines [39], but further research into community case management of neonates, especially in hard-to-access areas, is likely to offer a significant contribution to public health.

As many programmes have turned towards CHWs to increase coverage of their interventions, it has become clear that CHWs cannot do everything, especially if they are unpaid [34,40]. Detailed national and sub-national analysis is required to understand how integrated community-based approaches can be implemented in high mortality hard-to-access areas [38], including those affected by instability.

### Limitations

By its very nature, modelling entails simplifying reality to make useful predictions. Some important aspects of childhood pneumonia were excluded because they were not expected to have a significant impact on the relative effectiveness of the two strategies being compared. Certain unexplored issues may, however, have a more significant influence on the results. Sensitivity analysis was not conducted to explore the responsiveness of the outcome to changes in the median duration of disease, or how treatment adherence may differ between mobile clinics and fixed health posts. Analysis of alternative models of care-seeking behaviour revealed the possibility that mobile clinics may be the dominant strategy when attendance is very high and the mobile clinic is able to consult all patients who attend. In practice, mobile clinics may not be able to see all patients and so the degree to which these findings hold true will depend on the effectiveness of triage. Insecurity or other constraints may also cause a mobile team to miss planned visits, which, in addition to reducing this strategy's impact, may potentially also have untoward consequences for care-seeking practices, although evidence on this latter point is not currently available. Similarly, we assumed that CHWs would be available 100% of the time, and did not model the potential repercussions of the CHW running out of antibiotics or being unavailable. Estimating the true effect of either intervention would also require considering simultaneous diagnosis (or misdiagnosis) and treatment of co-morbidities, such as malaria.

Standard errors used for sensitivity analysis were estimated somewhat crudely and may therefore have underestimated the uncertainty in the results. All children began the model in the healthy state and a burn-in period was not used, which likely biased the expected number of child deaths downwards. While sensitivity analysis was conducted to examine the impact on results of different pneumonia incidences, outcomes were not examined in contexts of heightened vulnerability, such as would accompany high malnutrition or HIV rates, where the rate of disease progression and the CFR would increase [41]. Perhaps most influentially, the model does not measure the negative impact of unnecessary antibiotic treatment, incorrect prescriptions, and poor adherence on the individual child or on the wider community, particularly with respect to the development of resistant bacterial strains. Lim provides an example of how such negative outcomes can be included in a decision tree [42].

Like most studies, this analysis did not formally address methodological and model uncertainty, which Brisson and Edmunds have shown can play an even greater role than parameter uncertainty [43]. Parameter estimates themselves were based on a non-systematic review of the literature and the Delphi method was not used to elicit expert opinion on parameter values, although this method also has drawbacks [44]. The number of relevant studies with quantitative data on certain topics, such as care-seeking behaviour and treatment adherence in crises, was limited. While some grey literature was obtained, studies and reports, particularly from within the humanitarian community may have been missed. A broader limitation of this project is the lack of rigorous data collected by humanitarian agencies and ministries of health working in hard-to-reach areas.

## Conclusions

This paper's findings indicate that to reduce high under 5 mortality rates through curative care in hard-to-access populations, humanitarian health agencies, donors, and ministries of health should work together to:

• ensure that mobile clinic strategies explicitly consider how the timing of community visits, care-seeking behaviour, the type of intervention (preventive, curative for slow-onset illness, curative for rapid-onset illness), and other key variables identified in this model can be expected to influence the strategy's health impact;

• consider how training and support of CHWs can be integrated into the activities of a mobile team with high-level technical skills and resources;

• advocate for and design policies and programmes that enable CHWs to provide antibiotics at the community level;

• use models to guide health policy and explicit decision-making, especially for hard-to-access areas where current evidence is limited;

• prioritise research into care-seeking behaviour, treatment adherence, rates of disease progression and other key factors in service delivery strategies.

## List of abbreviations

(CFR): Case-fatality ratio; (CHW): Community health worker; (CI): Credible interval; (FCHV): female community health volunteers; (MDG): Millennium development goal.

## Competing interests

The authors declare that they have no competing interests.

## Authors' contributions

CP designed the study, developed the model, carried out analyses and co-wrote the paper. BR supervised the study and co-wrote the paper. FC designed and supervised the study and co-wrote the paper. All authors read and approved the final manuscript.

## Pre-publication history

The pre-publication history for this paper can be accessed here:

http://www.biomedcentral.com/1472-6963/12/9/prepub

## Supplementary Material

Additional file 1 **Appendix**. Appendix containing details on the model. Click here for file
