# Supplementary material for: Treating childhood pneumonia in hard-to-reach areas: A model-based comparison of mobile clinics and community-based care
Source: BMC Health Serv Res. 2012 Jan 10;12:9. doi: 10.1186/1472-6963-12-9 (PMC3276416; doi:10.1186/1472-6963-12-9)
Supplement: Additional file 1 — Appendix. Appendix containing details on the model. [file 1472-6963-12-9-S1.DOCX]

**Treating childhood pneumonia in hard-to-reach areas: A model-based comparison of mobile clinics and community-based care**

Catherine Pitt, Bayard Roberts, Francesco Checchi

**Appendix**

**Full model structure**

Figure 7 presents the full model structure, including the tunnel states used to incorporate temporary memory into the model. The simplified version of this structure is presented as Figure 1 in the main paper.

**Decision trees and transition matrix**

The following additional decision trees are presented:

- Transitions from the non-severe pneumonia state (Figure 8). If treatment is available and the child’s caregiver seeks treatment, the child transitions to the non-severe treatment state for the following three days. If treatment is not available or if the child’s caregiver does not seek care, the child faces the possibility of recovering, remaining non-severe, or developing severe pneumonia on the next day.
- Transitions from the non-severe under treatment state (Figure 9). All children who enter the treatment state remain in it for three days, after which time they transition to a new health state. While standard treatment may last five days, a caregiver may return to the care provider after three days if the child’s condition has worsened or not improved, and so three days was considered an appropriate duration for the model. After the three days, children who are correctly diagnosed and prescribed treatment, whose caregiver adheres to the treatment prescribed, and who are cured by the treatment transition to the healthy state. All other children transition to non-severe or severe pneumonia.
- Transitions from the severe pneumonia state (Figure 10), as above except that the possible transitions are to healthy state, severe pneumonia under treatment or death.
- Transitions from the severe pneumonia under treatment state (Figure 11), as above except that the possible transitions after three days of treatment are to healthy state, severe pneumonia or death.

Table 2 summarises the various decision trees as a single Markov matrix for the model. Rows represent the six possible health states in which a child may be at a given day, while columns represent the same six possible health states in which a child may be on the following day. Shaded cells indicate transitions that are not possible. For potential transitions, the symbol *π_x🡪y_* indicates the probability of transition from the current health state, x, to the next health state, y. In each row, the values of these probabilities *π_x🡪y_* sum to one.

**Calculation of transition probabilities**

Tables 3 and 4 detail parameter values and equations for care seeking and treatment availability. Tables 5 to 9 detail parameter values and equations for transition probabilities from the various states.

While some of the intermediate probabilities in the model could be estimated directly, others required converting a cumulative probability to a daily rate, and this rate to a daily probability. Generally, cumulative probabilities were converted to daily rates by rearranging the survival function and incorporating the cumulative risk and median duration as follows:

Survival function: S(t)=e^-λt^, where λ=event rate, and t=time duration (days)

Cumulative probability of event: 1-R(t)=S(t), where R(t) is the cumulative risk over a period t

Therefore, , where δ=median duration (in days) before the event

Rates were then transformed into daily probabilities as p=1 - e^-λ^.

Equations thus assume that the event rate is constant with respect to time and so the survival function takes an exponential form. In practice, the rate of disease progression or recovery is not actually constant with respect to time, as there is often a small population subset that progresses extremely quickly, while for most children the lowest rate of progression occurs in the early days of disease and increases thereafter (Brian Greenwood, personal communication). Nonetheless, such fluctuations in the rate of progression are unlikely to affect any differences observed in the effectiveness of mobile clinics compared with CHWs, and data are not currently available to support the modelling of such time-dependent rates. For these reasons, progression probabilities have been modelled with constant rates.

**Uncertainty distributions for parameter values**

The significant uncertainty regarding the true mean parameter values was captured through probabilistic sensitivity analysis (PSA). Two types of distributions were used, the beta and the lognormal. In both cases, the distributions were selected following the standard methods proposed by Briggs *et al* [1], ensuring that the distributions reflect our beliefs about the parameter and are defined over an appropriate interval. The beta distribution is the conjugate of the binomial distribution and represents the probability distribution of a proportion, bounded on the interval 0 to 1. It is therefore the appropriate distribution for dichotomous probabilities, including the daily risk of developing pneumonia [1]. Characterised by the parameters α and β, the beta distribution can be fitted to a given mean and standard deviation with the method of moments:

Method of moments: $\bar{\mu}=\frac{\alpha}{\alpha+\beta}$

$s^{2}=\frac{\alpha\beta}{\left(\alpha+\beta\right)^{2}\left(\alpha+\beta+1\right)}$

Solving for α and β,

For the median durations used to transform cumulative risks into daily rates, a lognormal distribution is most appropriate, as it is bounded on the interval 0 to infinity. While it is not clear that the distribution of the median would necessarily display the skew of the lognormal distribution, such skew is possible, while a simple normal distribution would allow the duration parameters to take on negative values, which is impossible.

In general, the standard deviation for a parameter value was estimated as five percent of the mean value of that parameter [2]. In all cases, parameters have been considered to vary independently from one another, except where one is clearly defined as a function of another.

**Figure 7. Full model structure.**

Healthy

Death

Non-severe day 1

Severe

day 1

Non-severe

treatment day 1

Severe treatment day 1

Severe treatment day 3

Severe treatment day 2

Severe

day 2

Severe

day 3

Severe

day 4

Severe

day 5+

Non-severe

treatment day 2

Non-severe

treatment day 3

Non-severe day 2

Non-severe day 3

Non-severe day 4

Non-severe day 5+

= Healthy state (H)

= Non-severe pneumonia state (N)

= Severe pneumonia state (S)

= Death state (D)

= Direction of possible

transition on next day

= Possibility of

remaining in the same

health state in next day

**KEY**

= Non-severe pneumonia treatment state

= Severe pneumonia treatment state

**Figure 8. Decision tree: transitions from the non-severe pneumonia state**

Non-severe

Does not

seek

health care

Healthy

Non-severe

Severe

Seeks

health care

Decision process

Health state

on current day

Health state

on next day

Health care is not available

Health care

is available

Non-severe treatment

Reverts to

no treatment outcome

**Figure 9. Decision tree: transitions from the non-severe pneumonia under treatment state**

Not receive correct treatment

Non-severe

treatment

Healthy

Non-severe

Severe

Correct diagnosis and treatment prescribed

Incorrect treatment prescribed

Adheres

to treatment

Does

not adhere

to treatment

Reverts to

no treatment outcome

Treatment

fails

Treatment cures

Decision process

Health state

on current day

Health state

After **3** days

Remains under treatment for non-severe pneumonia for 3 days

Death

**Figure 10. Decision tree: transitions from the severe pneumonia state**

Severe

Does not

seek

health care

Healthy

Severe

Death

Seeks

health care

Decision process

Health state

on current day

Health state

on next day

Health care is not available

Health care

is available

Severe treatment

Reverts to

no treatment outcome

**Figure 11. Decision tree: transitions from the severe pneumonia under treatment state**

Not receive correct treatment

Severe

treatment

Healthy

Severe

Death

Correct diagnosis and treatment prescribed

Incorrect treatment prescribed

Adheres

to treatment

Does

not adhere

to treatment

Reverts to

no treatment outcome

Treatment

fails

Treatment cures

Decision process

Health state

on current day

Health state

After **3** days

Remains under treatment for severe pneumonia for 3 days

Non-severe

**Table 2. Markov transition probabilities matrix.**

Each cell in the table represents the probability of transition from the health state indicated in the row to that indicated in the column. Shaded cells represent impossible transitions. Probabilities in each row sum to 1. For children receiving treatment for non-severe or severe pneumonia, the probability of remaining under treatment is 100% for three days, after which the child transitions to one of the remaining health states in the row.

|  |  | | **Health state on next day** | | | | | | | | | | | | | | | | | |
| --- | --- | --- | --- | --- | --- | --- | --- | --- | --- | --- | --- | --- | --- | --- | --- | --- | --- | --- | --- | --- |
|  |  | | **Healthy** | **Non-severe pneumonia** | | | | | **Non-severe under treatment** | | | **Severe pneumonia** | | | | | **Severe under treatment** | | | **Death** |
|  |  |  |  | **Day 1** | **Day 2** | **Day 3** | **Day 4** | **Day 5+** | **Day 1** | **Day 2** | **Day 3** | **Day 1** | **Day 2** | **Day 3** | **Day 4** | **Day 5+** | **Day 1** | **Day 2** | **Day 3** |  |
| **Health state on current day** | **Healthy** | | *π_H🡪H_* | *π_H🡪N_* |  |  |  |  | *π_H🡪NTreat_* |  |  | *π_H🡪S_* |  |  |  |  | *π_H🡪STreat_* |  |  |  |
|  | **Non-severe pneumonia** | **Day 1** | *π_N1🡪H_* |  | *π_N1🡪N_* |  |  |  | *π_N1🡪NTreat_* |  |  |  | *π_N1🡪S_* |  |  |  | *π_N1🡪STreat_* |  |  |  |
|  |  | **Day 2** | *π_N2🡪H_* |  |  | *π_N2🡪N_* |  |  | *π_N2🡪NTreat_* |  |  |  |  | *π_N2🡪S_* |  |  | *π_N2🡪STreat_* |  |  |  |
|  |  | **Day 3** | *π_N3🡪H_* |  |  |  | *π_N3🡪N_* |  | *π_N3🡪NTreat_* |  |  |  |  |  | *π_N3🡪S_* |  | *π_N3🡪STreat_* |  |  |  |
|  |  | **Day 4** | *π_N4🡪H_* |  |  |  |  | *π_N4🡪N_* | *π_N4🡪NTreat_* |  |  |  |  |  |  | *π_N4🡪S_* | *π_N4🡪STreat_* |  |  |  |
|  |  | **Day 5+** | *π_N5🡪H_* |  |  |  |  | *π_N5🡪N_* | *π_N5🡪NTreat_* |  |  |  |  |  |  | *π_N5🡪S_* | *π_N5🡪STreat_* |  |  |  |
|  | **Non-severe under treatment** | **Day 1** |  |  |  |  |  |  |  | 1 |  |  |  |  |  |  |  |  |  |  |
|  |  | **Day 2** |  |  |  |  |  |  |  |  | 1 |  |  |  |  |  |  |  |  |  |
|  |  | **Day 3** | *π_NTreat🡪H_* |  |  |  |  | *π_NTreat🡪N_* |  |  |  |  |  |  |  | *π_NTreat🡪S_* |  |  |  | *π_NTreat🡪D_* |
|  | **Severe pneumonia** | **Day 1** | *π_S1🡪H_* |  |  |  |  |  |  |  |  |  | *π_S1🡪S_* |  |  |  | *π_S1🡪STreat_* |  |  | *π_S1🡪D_* |
|  |  | **Day 2** | *π_S2🡪H_* |  |  |  |  |  |  |  |  |  |  | *π_S1🡪S_* |  |  | *π_S2🡪STreat_* |  |  | *π_S2🡪D_* |
|  |  | **Day 3** | *π_S3🡪H_* |  |  |  |  |  |  |  |  |  |  |  | *π_S1🡪S_* |  | *π_S3🡪STreat_* |  |  | *π_S3🡪D_* |
|  |  | **Day 4** | *π_S4🡪H_* |  |  |  |  |  |  |  |  |  |  |  |  | *π_S1🡪S_* | *π_S4🡪STreat_* |  |  | *π_S4🡪D_* |
|  |  | **Day 5+** | *π_S5🡪H_* |  |  |  |  |  |  |  |  |  |  |  |  | *π_S1🡪S_* | *π_S5🡪STreat_* |  |  | *π_S5🡪D_* |
|  | **Severe under treatment** | **Day 1** |  |  |  |  |  |  |  |  |  |  |  |  |  |  |  | 1 |  |  |
|  |  | **Day 2** |  |  |  |  |  |  |  |  |  |  |  |  |  |  |  |  | 1 |  |
|  |  | **Day 3** | *π_STreat🡪H_* |  |  |  |  | *π_STreat🡪N_* |  |  |  |  |  |  |  | *π_Streat🡪S_* |  |  |  | *π_STreat🡪D_* |
|  | **Death** | |  |  |  |  |  |  |  |  |  |  |  |  |  |  |  |  |  | 1 |

**Table 3. Parameters: care-seeking behaviour**

|  | **All Health States** | **Parameters applicable to both**  **mobile clinic and CHW scenarios** | | | |  |
| --- | --- | --- | --- | --- | --- | --- |
| **Symbol** | **Variable** | **Distribution** | **SD** | | **Mean** | **Source** |
| R_seek_ | Cumulative probability of seeking treatment | Beta | 0.045 | | 0.9 | Kallander *et al* [3]  Sodemann et al [4] |
| δ_seek_ | Median duration of illness episode before care sought (days) | Lognormal | n/a | | μ = 3.0, σ = 0.1 | Kallander *et al* [3]  Sodemann et al [4] |
| λ_seek_ | Average daily probability of seeking care |  | | | |  |
| RR_seek.i_ | Relative daily probability of seeking care (compared to the average probability: the subscripted number indicates the day since illness onset) | RR_seek.1_ = 1  RR_seek.2_ = 1.3  RR_seek.3_ = 1.6 | | RR_seek.4_ = 1.2  RR_seek.5+_ = 0.8 | | Derived from data on the duration of illness before seeking treatment outside the home in fatal cases in Kallander *et al* [3] and Sodemann *et al*[4] |
| p_seek.i_ | Time-dependent probability that case will seek treatment if it's available, for any day i | $1-e^{-\lambda_{seek}*RR_{seek.i}}$ Sensitivity analysis range for mobile clinics only: 0.25, 0.5, 0.75, 1.0 | | | |  |

**Table 4. Parameters: frequency of mobile clinic visits**

| **Variable** | **Value** | **Source** |
| --- | --- | --- |
| Frequency of mobile clinic visits to the community | Every 7 days  Sensitivity analysis: Every 1-10, 14, 21 or 28 days | Du Mortier and Coninx [5] |

**Table 5. Parameters: transition probabilities from the healthy state**

|  | **HEALTHY** | **Parameters applicable to**  **no treatment, mobile, and CHW scenarios** | | |  |
| --- | --- | --- | --- | --- | --- |
| **Symbol** | **Variable** | **Distribution** | **SD** | **Mean** | **Source** |
| λ_H🡪disease_ | Disease incidence (daily rate per child) | Beta | 0.0001 | 0.001945  Sensitivity range:  0.000822 - 0.006301 | Baseline taken from the 75^th^ percentile in Rudan’s review of the developing world [6], supported by other site-specific studies [7-10] |
| p_H🡪disease_ | Daily probability of transition to disease (both severe and non-severe) | $1-e^{-\lambda_{H\to disease}}$ | | |  |
| p_S/disease_ | Proportion of all pneumonia cases that are severe on the first day | Beta | 0.005 | 0.05 |  |
| *π*_H🡪H_ | Daily probability of remaining healthy (no treatment available) | $e^{-\lambda_{H\to disease}}$ | | |  |
| p_H🡪N_ | Daily probability of transition to non-severe pneumonia | p_H🡪disease_ * (1 - p_S/disease_) | | |  |
| p_H🡪S_ | Daily probability of transition to severe pneumonia | p_H🡪disease_ * p_S/disease_ | | |  |
| *π*_H🡪NTreat_ | Daily probability of transition to non-severe treatment (treatment available) | p_H🡪N_ * p_N.available_ * (1 – e^-λseek.1^) | | |  |
| *π*_H🡪STreat_ | Daily probability of transition to severe treatment (treatment available) | p_H🡪S_ * p_S.available_ * (1 – e^-λseek.1^) | | |  |
| *π*_H🡪N_ | Daily probability of transition to non-severe no treatment (treatment available) | p_H🡪N_ * (1 – (p_N.available_ * (1 – e^-λseek.1^))) | | |  |
| *π*_H🡪S_ | Daily probability of transition to severe no treatment (treatment available) | p_H🡪S_ * (1 – (p_S.available_ * (1 – e^-λseek.1^))) | | |  |

**Table 6. Parameters: transition probabilities from the non-severe pneumonia state**

|  | **NON-SEVERE (without treatment)** | **Parameters applicable to**  **no treatment, mobile, and CHW scenarios** | | | |  |
| --- | --- | --- | --- | --- | --- | --- |
| **Symbol** | **Variable** | **Distribution** | **Mean** | | **SD** | **Source** |
| δ_N🡪H_  δ_N🡪S_ | Median illness duration, either resulting in recovery to the healthy state or transition to severe pneumonia without treatment (days) | Lognormal | 3 | | 0.3 | Kallander *et al* [3] |
| R_N🡪H_ | Cumulative probability of transition to healthy (recovery) without treatment | Beta | 0.90 | | 0.025 | Rudan *et al*[6] |
| R_N🡪S_ | Cumulative probability of transition to severe without treatment | 1 - R_N🡪H_ | | | |  |
| λ_N🡪H_ | Daily rate of transition to healthy without treatment |  | | | |  |
| λ_N🡪S_ | Daily rate of transition to severe without treatment |  | | | |  |
| p_N🡪H_ | Daily probability of transition to healthy without treatment | 1 - e ^- λN🡪H^ | | | |  |
| p_N🡪S_ | Daily probability of transition to severe without treatment | 1 - e ^- λN🡪S^ | | | |  |
| p_N🡪N_ | Daily probability of remaining non-severe without treatment | 1 - p_N🡪H_ - p_N🡪S_ | | | |  |
| p_N.available_ | Probability that treatment is available on any given day | Mobile:  If present: 1  If not present: 0 | | CHW: 1 | |  |
| *π*_Ni🡪NTreat_ | Daily probability of transition to treatment for non-severe pneumonia (treatment available) | p_N.available_ * p_seek.i_ | | | |  |
| *π*_Ni🡪H_ | Daily probability of transition to healthy without treatment (treatment available) | (1 - p_N.available_ * p_seek.i_) * p_N🡪H_ | | | |  |
| *π*_Ni🡪S_ | Daily probability of transition to severe without treatment (treatment available) | (1 - p_N.available_ * p_seek.i_) * p_N🡪S_ | | | |  |
| *π*_Ni🡪N_ | Daily probability of remaining non-severe without treatment (treatment available) | (1 - p_N.available_ * p_seek.i_) * p_N🡪N_ | | | |  |

**Table 7. Parameters: transition probabilities from the non-severe pneumonia under treatment state**

|  | **NON-SEVERE UNDER TREATMENT** | **Parameters** | | |  |
| --- | --- | --- | --- | --- | --- |
| **Symbol** | **Variable** | **Distribution** | **Mean** | **SD** | **Source** |
| p_N.correct_ | Cumulative probability that case accessing treatment is correctly diagnosed and prescribed | Beta | Mobile: 0.9  Fixed: 0.8 | Mobile: 0.04  Fixed: 0.03 | Kallander *et al*[11]  Dawson *et al*[12] |
| p_N.adhere_ | Probability of adherence to treatment | Beta | 0.8 | 0.08 | Checchi *et al[13]* |
| p_N.cure_ | Probability of treatment curing case if child’s caregiver adheres to prescription | Beta | 0.95 | 0.0475 | Hazir *et al***[14]**  Lim *et al*[15] |
| p_N🡪H3_ | Probability of transition from non-severe to healthy after three days without treatment | (p_N🡪H_ * p_H🡪H_ * p_H🡪H_ )+ (p_N🡪H_ * p_H🡪N_ * p_N🡪H_ )+ (p_N🡪H_ * p_H🡪H_ * p_S🡪H_ )+ (p_N🡪N_ * p_N🡪H_ * p_H🡪H_ )+ (p_N🡪N_ * p_N🡪N_ * p_N🡪N_ )+ (p_N🡪N_ * p_N🡪N_ * p_N🡪N_ )+ (p_N🡪S_ * p_N🡪N_ * p_N🡪N_ )+ (p_N🡪S_ * p_N🡪N_ * p_N🡪N_) | | |  |
| p_N🡪N3_ | Probability of remaining non-severe after three days without treatment | (p_N🡪H_ * p_H🡪H_ * p_H🡪N_ )+ (p_N🡪H_ * p_H🡪N_ * p_N🡪H_ )+ (p_N🡪N_ * p_N🡪H_ * p_H🡪H_ )+ (p_N🡪N_ * p_N🡪N_ * p_N🡪H_ )+ (p_N🡪S_ * p_S🡪H_ * p_H🡪H_ ) | | |  |
| p_N🡪S3_ | Probability of transition from non-severe to severe after three days without treatment | (p_N🡪H_ * p_H🡪H_ * p_H🡪S_ )+ (p_N🡪H_ * p_H🡪N_ * p_N🡪S_ )+ (p_N🡪H_ * p_H🡪S_ * p_S🡪S_ )+ (p_N🡪N_ * p_N🡪H_ * p_H🡪S_ )+ (p_N🡪N_ * p_N🡪N_ * p_N🡪S_ )+ (p_N🡪N_ * p_N🡪S_ * p_S🡪S_ )+ (p_N🡪S_ * p_S🡪H_ * p_H🡪S_ )+ (p_N🡪S_ * p_S🡪S_ * p_S🡪S_ ) | | |  |
| p_N🡪D3_ | Probability of transition from non-severe to death after three days without treatment | (p_N🡪H_ * p_H🡪S_ * p_S🡪D_ )+ (p_N🡪N_ * p_N🡪S_ * p_S🡪D_ )+ (p_N🡪S_ * p_S🡪S_ * p_S🡪D_ )+ (p_N🡪H_ * p_S🡪D_ * p_D🡪D_ ) | | |  |
| *π*_NTreat🡪H_ | Probability of transition to healthy after 3 days of treatment | p_N.correct_ *p_N.adhere_ *(p_N.cure_ – p_N🡪H3_) + p_N🡪H3_ | | |  |
| *π*_NTreat🡪N_ | Probability of remaining non-severe after 3 days of treatment | p_N🡪N3_ * (1- *π*_NTreat🡪H_) / (1 - p_N🡪H3_) | | |  |
| *π*_NTreat🡪S_ | Probability of transition to severe after 3 days of treatment | p_N🡪S3_ * (1- *π*_NTreat🡪H_) / (1 - p_N🡪H3_) | | |  |
| *π*_NTreat🡪D_ | Probability of transition to death after 3 days of treatment | p_N🡪D3_ * (1- *π*_NTreat🡪H_) / (1 - p_N🡪H3_) | | |  |

**Table 8. Parameters: Transition probabilities from the severe pneumonia state**

|  | **SEVERE (without treatment)** | **Parameters applicable to**  **no treatment, mobile, and CHW scenarios** | | | |  |
| --- | --- | --- | --- | --- | --- | --- |
| **Symbol** | **Variable** | **Distribution** | **Mean** | | **SD** | **Source** |
| δ_S🡪H_  δ_S🡪D_ | Median illness duration, either resulting in recovery to the healthy state or death (days) | Lognormal | 3 | | 0.1 | Hazir *et al*[14]  Kallander *et al* [3] |
| R_S🡪H_ | Cumulative probability of transition to healthy without treatment | Beta | 0.75 | | 0.0375 | Derived by combining age-specific CFR in pre-antibiotic era in Mulholland [16] with proportion of pneumonia by age in Rudan [6];  consistent with Lim *et al* [15] |
| R_S🡪D_ | Cumulative probability of death without treatment (CFR) | 1 – R_S🡪H_ | | | |  |
| λ_S🡪H_ | Daily rate of transition to healthy without treatment |  | | | |  |
| λ_S🡪D_ | Daily rate of death without treatment |  | | | |  |
| p_S🡪H_ | Daily probability of transition to healthy without treatment | 1 - e ^– λS🡪H^ | | | |  |
| p_S🡪D_ | Daily probability of death without treatment | 1 - e ^- λS🡪D^ | | | |  |
| p_S🡪S_ | Daily probability of remaining severe without treatment | 1 - p_N🡪H_ - p_N🡪D_ | | | |  |
| p_S.available_ | Probability that treatment is available on any given day | Mobile:  If present: 1  If not present: 0 | | CHW: 1 | |  |
| *π*_Si🡪STreat_ | Daily probability of transition to treatment for severe pneumonia (treatment available) | P_S.available_ * p_seek.i_ | | | |  |
| *π*_Si🡪H_ | Daily probability of transition to healthy without treatment (treatment available) | (1 – p_S.available_ * p_seek.i_) * p_S🡪H_ | | | |  |
| *π*_Si🡪D_ | Daily probability of death without treatment (treatment available) | (1 – p_S.available_ * p_seek.i_) * p_S🡪D_ | | | |  |
| *π*_Si🡪S_ | Daily probability of remaining severe without treatment (treatment available) | (1 – p_S.available_ * p_seek.i_) * p_S🡪S_ | | | |  |

**Table 9. Parameters: transition probabilities from the severe pneumonia under treatment state**

|  | **SEVERE UNDER TREATMENT** | **Parameters** | | | |  |
| --- | --- | --- | --- | --- | --- | --- |
|  | **Variable** | **Distribution** | **Mean** | **SD** | | **Source** |
| p_S.correct_ | Cumulative probability that case accessing treatment is correctly diagnosed and prescribed | Beta | Mobile: 0.9  Fixed: 0.8 | Mobile: 0.045  Fixed: 0.04 | | Kallander *et al* [11]  Hazir *et al*[14]  Dawson *et al* [12] |
| p_S.adhere_ | Probability of adherence to treatment | Beta | 0.8 | 0.08 | | Checchi *et al* [13] |
| p_S.cure_ | Probability of treatment curing case if child’s caregiver adheres to prescription | Beta | Mobile: 0.9  Fixed: 0.8 | Mobile: 0.045  Fixed: 0.04 | | Kabra *et al [17]*  Lim *et al* [15]  Zaman *et al* [8]  Johnson *et al* [18]  Hazir *et al*[14]  Banajeh *et al* [19] |
| p_S🡪H3_ | Probability of transition from severe to healthy after three days without treatment | (p_S🡪H_ * p_H🡪H_ * p_H🡪H_ )+ (p_S🡪H_ * p_H🡪N_ * p_N🡪H_ )+ (p_S🡪H_ * p_H🡪S_ * p_S🡪H_ )+ (p_S🡪S_ * p_S🡪H_ * p_H🡪H_ )+ (p_S🡪S_ * p_S🡪S_ * p_S🡪N_) | | | |  |
| p_S🡪N3_ | Probability of transition from severe to non-severe after three days without treatment | (p_S🡪H_ * p_H🡪H_ * p_H🡪N_ )+ (p_S🡪H_ * p_H🡪N_ * p_N🡪N_ )+ (p_S🡪S_ * p_S🡪H_ * p_H🡪N_) | | | |  |
| p_S🡪S3_ | Probability of remaining severe after three days without treatment | (p_S🡪H_ * p_H🡪H_ * p_H🡪S_ )+ (p_S🡪H_ * p_H🡪N_ * p_N🡪S_ )+ (p_S🡪H_ * p_H🡪S_ * p_S🡪S_ )+ (p_S🡪S_ * p_S🡪H_ * p_H🡪S_ )+ (p_S🡪S_ * p_S🡪S_ * p_S🡪S_) | | | |  |
| p_S🡪D3_ | Probability of transition from severe to death after three days without treatment | (p_S🡪H_ * p_H🡪S_ * p_S🡪D_ )+ (p_S🡪S_ * p_S🡪S_ * p_S🡪D_ )+ (p_S🡪S_ * p_S🡪D_ * p_D🡪D_ )+ (p_S🡪D_ * p_D🡪D_ * p_D🡪D_ ) | | | |  |
| *π*_STreat🡪H_ | Probability of transition to healthy after 3 days of treatment | p_S.correct_ *p_S.adhere_ *(p_S.cure_ – p_S🡪H3_) + p_S🡪H3_ | | | |  |
| *π*_STreat🡪N_ | Probability of transition to non-severe after 3 days of treatment | p_S🡪N3_ * (1- *π*_STreat🡪H_) / (1 - p_S🡪H3_) | | |  | |
| *π*_STreat🡪S_ | Probability of remaining severe after 3 days of treatment | p_S🡪S3_ * (1- *π*_STreat🡪H_) / (1 - p_S🡪H3_) | | |  | |
| *π*_STreat🡪D_ | Probability of transition to severe after 3 days of treatment | p_N🡪D3_ * (1- *π*_NTreat🡪H_) / (1 - p_N🡪H3_) | | |  | |

**References for the appendix**

1. Briggs AH, Claxton K, Sculpher MJ: *Decision modelling for health economic evaluation.* Oxford: Oxford University Press; 2006.

2. Critchfield GC, Willard KE: **Probabilistic analysis of decision trees using Monte Carlo simulation.** *Med Decis Making* 1986, **6:**85-92.

3. Kallander K, Hildenwall H, Waiswa P, Galiwango E, Peterson S, Pariyo G: **Delayed care seeking for fatal pneumonia in children aged under five years in Uganda: a case-series study.** *Bull World Health Organ* 2008, **86:**332-338.

4. Sodemann M, Jakobsen MS, Molbak K, Alvarenga IC, Jr., Aaby P: **High mortality despite good care-seeking behaviour: a community study of childhood deaths in Guinea-Bissau.** *Bull World Health Organ* 1997, **75:**205-212.

5. du Mortier S, Coninx R: **Mobile health units in emergency operations: a methodological approach.** In *Book Mobile health units in emergency operations: a methodological approach* (Editor ed.^eds.). City: Humanitarian Practice Network; 2007.

6. Rudan I, Tomaskovic L, Boschi-Pinto C, Campbell H: **Global estimate of the incidence of clinical pneumonia among children under five years of age.** *Bull World Health Organ* 2004, **82:**895-903.

7. Reddaiah VP, Kapoor SK: **Acute respiratory infections in rural underfives.** *Indian J Pediatr* 1988, **55:**424-426.

8. Zaman K, Baqui AH, Yunus M, Sack RB, Bateman OM, Chowdhury HR, Black RE: **Acute respiratory infections in children: a community-based longitudinal study in rural Bangladesh.** *J Trop Pediatr* 1997, **43:**133-137.

9. Smith TA, Lehmann D, Coakley C, Spooner V, Alpers MP: **Relationships between growth and acute lower-respiratory infections in children aged less than 5 y in a highland population of Papua New Guinea.** *Am J Clin Nutr* 1991, **53:**963-970.

10. Lehmann D: **Epidemiology of acute respiratory tract infections, especially those due to Haemophilus influenzae, in Papua New Guinean children.** *J Infect Dis* 1992, **165 Suppl 1:**S20-25.

11. Kallander K, Tomson G, Nsabagasani X, Sabiiti JN, Pariyo G, Peterson S: **Can community health workers and caretakers recognise pneumonia in children? Experiences from western Uganda.** *Trans R Soc Trop Med Hyg* 2006, **100:**956-963.

12. Dawson P, Pradhan Y, Houston R, Karki S, Poudel D, Hodgins S: **From research to national expansion: 20 years' experience of community-based management of childhood pneumonia in Nepal.** *Bull World Health Organ* 2008, **86:**339-343.

13. Checchi F, Gayer M, Grais R, Mills E: **Public health in crisis-affected populations: A practical guide for decision-makers, Network Paper 61.** In *Book Public health in crisis-affected populations: A practical guide for decision-makers, Network Paper 61* (Editor ed.^eds.), vol. Number 61. City: Humanitarian Practice Network at the Overseas Development Institute; 2007.

14. Hazir T, Fox LM, Nisar YB, Fox MP, Ashraf YP, MacLeod WB, Ramzan A, Maqbool S, Masood T, Hussain W, et al: **Ambulatory short-course high-dose oral amoxicillin for treatment of severe pneumonia in children: a randomised equivalency trial.** *Lancet* 2008, **371:**49-56.

15. Lim YW, Steinhoff M, Girosi F, Holtzman D, Campbell H, Boer R, Black R, Mulholland K: **Reducing the global burden of acute lower respiratory infections in children: the contribution of new diagnostics.** *Nature* 2006, **444 Suppl 1:**9-18.

16. Mulholland K: **Perspectives on the burden of pneumonia in children.** *Vaccine* 2007, **25:**2394-2397.

17. Kabra SK, Lodha R, Pandey RM: **Antibiotics for community acquired pneumonia in children.** *Cochrane Database Syst Rev* 2006, **3:**CD004874.

18. Johnson AW, Osinusi K, Aderele WI, Adeyemi-Doro FA: **Bacterial aetiology of acute lower respiratory infections in pre-school Nigerian children and comparative predictive features of bacteraemic and non-bacteraemic illnesses.** *J Trop Pediatr* 1993, **39:**97-106.

19. Banajeh SM: **Outcome for children under 5 years hospitalized with severe acute lower respiratory tract infections in Yemen: a 5 year experience.** *J Trop Pediatr* 1998, **44:**343-346.
